# A Small Mammal Community in a Forest Fragment, Vegetation Corridor and Coffee Matrix System in the Brazilian Atlantic Forest

**DOI:** 10.1371/journal.pone.0023312

**Published:** 2011-08-31

**Authors:** Mariana Ferreira Rocha, Marcelo Passamani, Júlio Louzada

**Affiliations:** Setor de Ecologia, Departamento de Biologia, Universidade Federal de Lavras, Lavras, Brazil; University of California, Berkeley, United States of America

## Abstract

The objective of our work was to verify the value of the vegetation corridor in the conservation of small mammals in fragmented tropical landscapes, using a model system in the southeastern Minas Gerais. We evaluated and compared the composition and structure of small mammals in a vegetation corridor, forest fragments and a coffee matrix. A total of 15 species were recorded, and the highest species richness was observed in the vegetation corridor (13 species), followed by the forest fragments (10) and the coffee matrix (6). The absolute abundance was similar between the vegetation corridor and fragments (F = 22.94; p = 0.064), and the greatest differences occurred between the vegetation corridor and the matrix (F = 22.94; p = 0.001) and the forest fragments and the matrix (F = 22.94; p = 0.007). Six species showed significant habitat preference possibly related to the sensitivity of the species to the forest disturbance. *Marmosops incanus* was the species most sensitive to disturbance; *Akodon montensis*, *Cerradomys subflavus*, *Gracilinanus microtarsus* and *Rhipidomys* sp. displayed little sensitivity to disturbance, with a high relative abundance in the vegetation corridor. *Calomys* sp. was the species least affected by habitat disturbance, displaying a high relative abundance in the coffee matrix. Although the vegetation corridors are narrow (4 m width), our results support the hypothesis in which they work as a forest extension, share most species with the forest fragment and support species richness and abundance closer to forest fragments than to the coffee matrix. Our work highlights the importance and cost-effectiveness of these corridors to biodiversity management in the fragmented Atlantic Forest landscapes and at the regional level.

## Introduction

The deforestation of the Brazilian Atlantic Forest over the last two centuries has resulted in a highly fragmented landscape [1]. Except for a few large (>10.000 ha) governmental preserves, most of the 8% remaining Atlantic Forest is composed of small isolated patches of secondary forests (<80 ha) immersed in agricultural or occasionally urban matrices [2]. In the last two decades, studies of the Atlantic Forest fragments have demonstrated that landscape changes have drastic ecological consequences on small mammals communities, reducing the incidence of forest specialists and increasing generalist species in the smallest fragments [3], [4], [5], [6].

Small mammal species displaying low colonization and dispersion abilities in relation to their surrounding matrix environment are more vulnerable to the deleterious effects of fragmentation, reducing their persistence in fragmented landscapes [4], [7], [8], [9], [10]. Therefore, the persistence of small mammals in fragmented landscapes is associated with the functional connectivity of the landscape [4], [7], [8], [9], provided by structural components such as hedgerows, forest strips, riparian corridors and forested “terra firme” corridors [11], [12], [13].

Forest corridors are important components of landscape structure and function, especially where less permeable matrices predominate [11], [14]. By allowing species to disperse and colonize fragments, vegetation corridors help maintain the richness, composition and abundance of small mammals in agricultural landscapes [12], [15], [16], [17], [18], [19], increasing the functional connectivity in fragmented landscapes [11], [12], [14]. Vegetation corridors also increase the movement of individuals between fragments [19], [20], [21] and may serve as a habitat for many species of mammals [12], [15], [18], [22].

Most of the landscape in the south and southwest of Minas Gerais, Brazil, is composed of small forest fragments immersed in an agriculturally diversified matrix. Although vegetation corridors occur frequently, they are far less studied than the remaining fragments. In this study, we evaluated the conservation value of thin forest strips (corridors) resulting from the tree colonization of linear ditches. These ditches were made by the slave workforce during the nineteenth century to isolate pastures and farm borders. We evaluated the composition and structure of the small mammal community in forest fragments, a vegetation corridor and a coffee matrix in a model system.

## Methods

### Study area and sample sites

**Figure 1:**
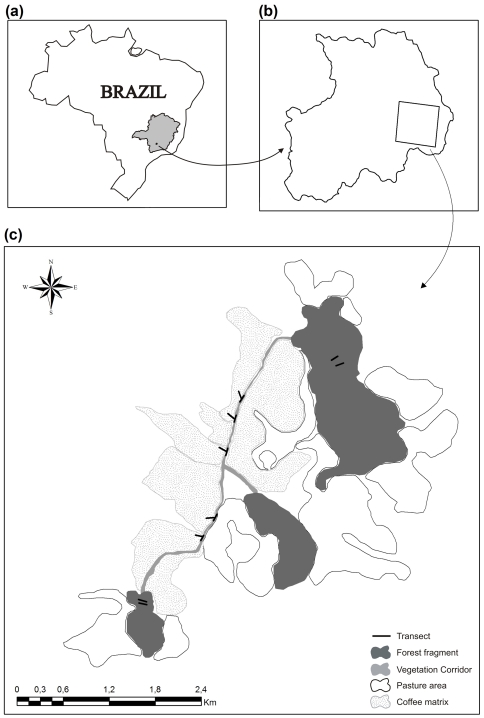
(A) Location of the municipality of Santo Antônio do Amparo, Minas Gerais, Brazil. (B) Location of the study area. (C) Schematic design of the treatments and transects.

The study area consists of two forest fragments and a vegetation corridor immersed in a coffee matrix, located in the municipality of Santo Antônio do Amparo, Minas Gerais, Brazil (20°53′57.1″S, 44°50′11.5″W, Figure 1). The climate of this region is classified as Cwa (humid climate with dry winter and hot summer), according to Koeppen, with an annual mean temperature of 19.9°C and annual mean precipitation of 1.597 mm [23], [24].

The vegetation is classified as semi-deciduous seasonal forest, with floristic influences of Cerrado [25], [26]. The most important families in the forest fragments and the vegetation corridor included Fabaceae, Myrtaceae, Lauraceae, Rubiaceae, Annonaceae, Euphorbiaceae and Meliaceae. Floristically, the fragments were dominated by *Protium spruceanum*, *Copaifera langsdorffii*, *Myrcia splendens*, *Tapirira obtusa* and *Magnolia ovate*, and *Pera glabrata*, *Copaifera langsdorffii*, *Casearia arborea*, *Protium widgrenii* and *Tapirira obtuse* dominated the corridors [26]. The composition and abundance of woody species in the corridor were more similar to the interior than the edge of the fragments, and the diversity index values for the fragment and corridor (3.75 and 3.74, respectively) were among the largest found for other forest remnants in the region studied.

The forest fragments studied are 26 ha and 48 ha in size and were the few remnants remaining after the large scale introduction of coffee crops in the region. The vegetation corridor has historic and cultural importance because it occurs in ditches or excavations built by slaves in the late nineteenth century to divide pieces of property and was formed through the natural tree colonization of these ditches [26]. Their dimensions are from 1.5 to 2.5 m in depth, 4 m in width and 3.2 km in length, with a main axis linking two fragments of forest [26] fenced by a matrix consisting primarily of coffee plantations (*Coffea arabica* L.) that are 2 m in height and to a smaller degree by pastures. This system of fragments connected by corridors (Figure 1) allows the evaluation of how small mammals use the landscape.

### Sample design and data collection

The small mammals were sampled in two forest fragments, one vegetation corridor and one coffee matrix, referred to here as treatments. The peculiar features of each treatment, including shape and size, prevented us from acquiring an equal sample size from each treatment. Thus, in each forest fragment, two parallel transects 100 m in length and 50 m apart were marked. In the vegetation corridor, five transects 100 m in length were placed in lines approximately 250 m from each other. In the coffee matrix, five transects 100 m in length were installed perpendicular to the corridor transects. Thus, 14 transects in total were marked. This sampling design enabled us to evaluate which species were present in each treatment and to determine any species exclusive to certain treatments.

In each transect, six sampling stations were marked with a distance of 20 m from each other. Two traps were placed in each station, one on the ground (lower strata) and another in the understory vegetation at 1–2 m above the ground (middle strata). The ground trap alternated between the large (43.0×12.5×14.5 cm) and small (25.0×9.0×8.0 cm) Sherman traps and medium and large (45.0×16.0×16.0 cm) wire mesh traps. Only small Sherman traps were used in the understory vegetation.

Ten day sampling periods were performed monthly between December 2008 and May 2009, totaling 60 days of sampling at each site and an effort of 2.880 trap-nights in the fragments, 3.600 in the vegetation corridor and 3,600 in the matrix, with the total sampling effort of 10.080 trap-nights. We marked the captured individuals in one ear with a numbered tag (National Band and Tag Co.) and released them in the same place of capture to undertake capture/recapture data. We collected voucher specimens of all species, which were determined by specialists (Y.Leite, L.P. Costa, R.C. Duda, and J.A. de Oliveira) and were hostened to the collection of the Laboratory of Ecology and Mammals Conservation of the Universidade Federal de Lavras. All procedures regarding capture and tagging of animals were conducted under the legal approval and consent of the Brazilian Federal Authority (IBAMA process number 14083-1) and by following the guidelines of the American Society of Mammalogists [27].

### Data analyses

The total abundance, or sum of the individuals captured in every species, and the abundance per species, or number of individuals captured in each species, were calculated on a transect basis.

The observed richness of the species was compared among the sampled treatments by contrasting the individual-based rarefaction curve (Mao Tau estimation) generated using EstimateS 8.0 software [28] with 1.000 randomizations.

To compare the community composition and structure between the forest fragments, corridor and coffee matrix, multidimensional-scaling (MDS) ordinations were used with qualitative (composition) and quantitative (structure) data in a similarity matrix obtained using Bray-Curtis similarity matrices. Analysis of similarity (ANOSIM) were used to assess the differences between sampling treatments. These analyses were also used in other studies that evaluate community structure in different landscapes [29], [30].

The indicator species analysis was used for species that presented at least 15 captured individuals in all samples and verifies the fidelity of species in the treatments through their abundance and relative frequency, the results of which are described by the observed indication value [31]. Significance was calculated by the Monte Carlo permutation test, using p = 0.05.

## Results

### Richness and composition

We obtained 978 captures of 444 individuals belonging to 15 species, including seven marsupials (*Marmosops incanus*, *Gracilinanus microtarsus*, *Monodelphis iheringi*, *Monodelphis americana*, *Caluromys philander*, *Didelphis albiventris* and *Didelphis aurita*) and eight rodents (*Akodon montensis*, *Rhipidomys* sp., *Cerradomys subflavus*, *Euryoryzomys russatus*, *Calomys* sp., *Nectomys squamipes*, *Oligoryzomys nigripes* and *Oxymycterus delator*).

**Figure 2:**
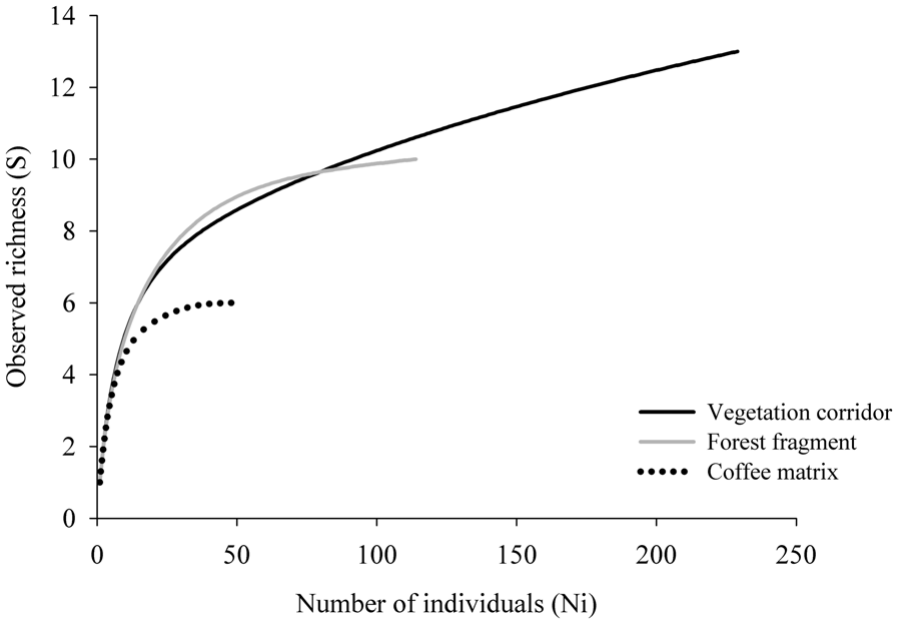
Rarefaction curve of the observed richness of small mammals in treatments.

The rarefaction curve indicates that in a standardized sampling effort of 60 individuals, contrasting differences exist between the richness of treatments (Figure 2).

The coffee matrix presented the least richness (6 species), stabilizing with approximately 30 individuals and indicating few active species in this system. The corridor richness (13) was greater than that of the fragment (10), and the curve did not stabilize, suggesting that the corridors harbor a larger number of species than the other sampled systems.

Few species were shared among the sampled treatments. Only three (*A. montensis*, *M. incanus* and *C. subfavus*) were common in all, and four occurred in only one. *N. squamipes* and *O. delator* were restricted to the fragment, and *M. americana* and *D. aurita* were exclusive to the corridor.

**Figure 3:**
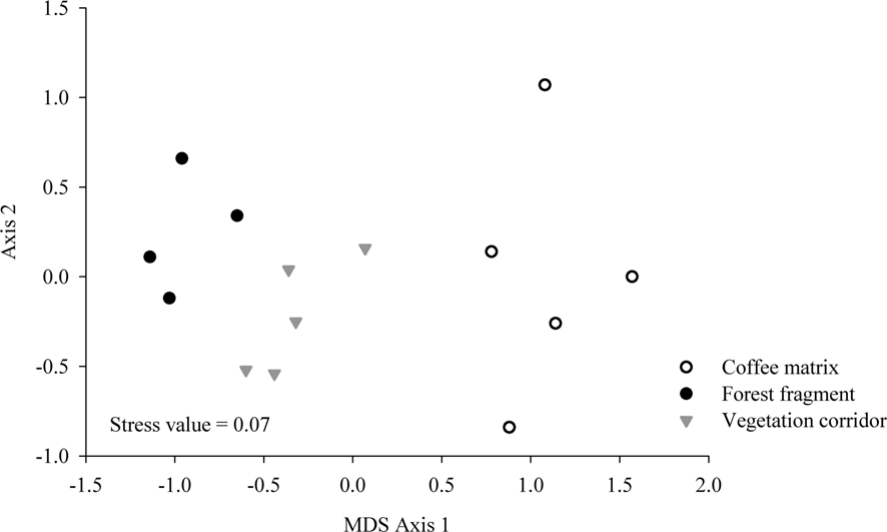
MDS analysis of the 14 sampled transects regarding species composition of small mammals in the fragment-corridor-matrix system.

The MDS analysis illustrated the low similarity in species composition among treatments, whith the dimensional separation of the three land uses (Figure 3) in three distinct compositional and structural groups. The ANOSIM confirmed these results, showing that the species composition differed significantly among the treatments (R global = 0.663, p = 0.001). The dissimilarity was greater between the corridor and the matrix (R = 0.762, p = 0.008) than between these and the fragment (R = 0.609, p = 0.008). The species composition was rather dissimilar between the fragment and the matrix (R = 0.916, p = 0.008).

### Disturbance-sensitive species and abundance analysis

**Figure 4:**
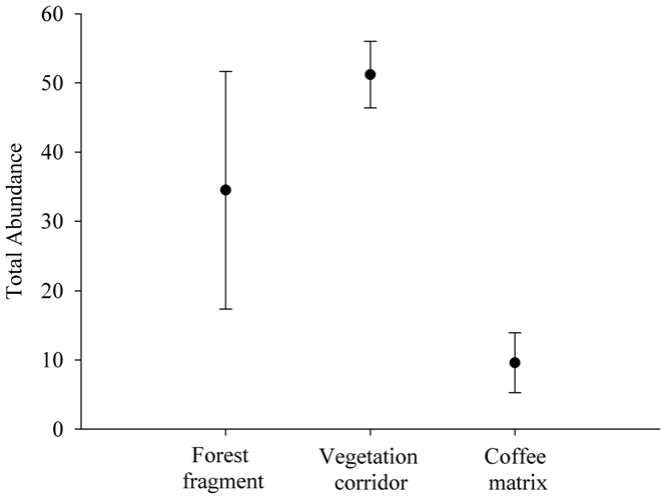
Mean and total standard deviation of small mammals in the forest fragment (FRAGM), corridor (CORR) and coffee matrix (MATR).

An average of 51.2 individuals were captured in the corridor, followed by 34.5 individuals in the fragment and 9.6 individuals in the matrix, indicating a statistically significant difference (F = 22.94, p = 0.001). However, the mean abundance was similar between the corridor and the fragment (F = 22.94; p = 0.064) and was significantly smaller in the matrix (F = 22.94; p = 0.007 between the matrix and fragment; F = 22.94; p = 0.001 between the matrix and the corridor) (Figure 4).

**Figure 5:**
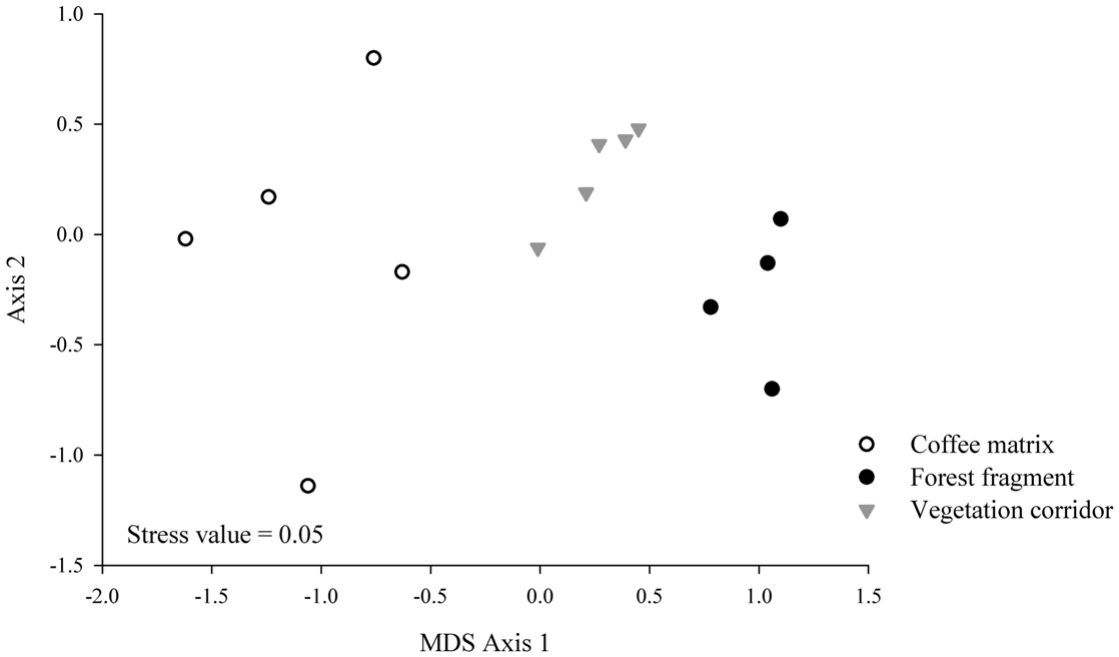
MDS analysis of the 14 transects sampled regarding species abundance of small mammals in the fragment-corridor-matrix system.

The MDS analysis illustrated differences between the three treatments (Figure 5), spatially separated as independent groups according the ANOSIM (R global = 0.782; p = 0.001). These results enhance the structural differences in small mammal communities active in the corridor, fragment and matrix (ANOSIM comparison: corridor×fragment: R = 0.913, p = 0.008; corridor×matrix: R = 0.78, p = 0.008; fragment×matrix: R = 0.956, p = 0.008).

The most abundant species in the fragment were *M. incanus* (50 individuals), *A. montensis* (32), *Rhipidomys* sp. (17) and *E. russatus* (12). In the corridor, these were also the most abundant rodents (77, 43 and 19 individuals, respectively), together with *C. subflavus* (23) and the marsupial *G. microtarsus* (52), which was not captured in the fragments. In the matrix, the most abundant species were *Calomys* sp. (15), *C. subflavus* (13) and *G. microtarsus* (10). The remaining species were less abundant with less than 10 individuals captured in all treatments.

**Figure 6:**
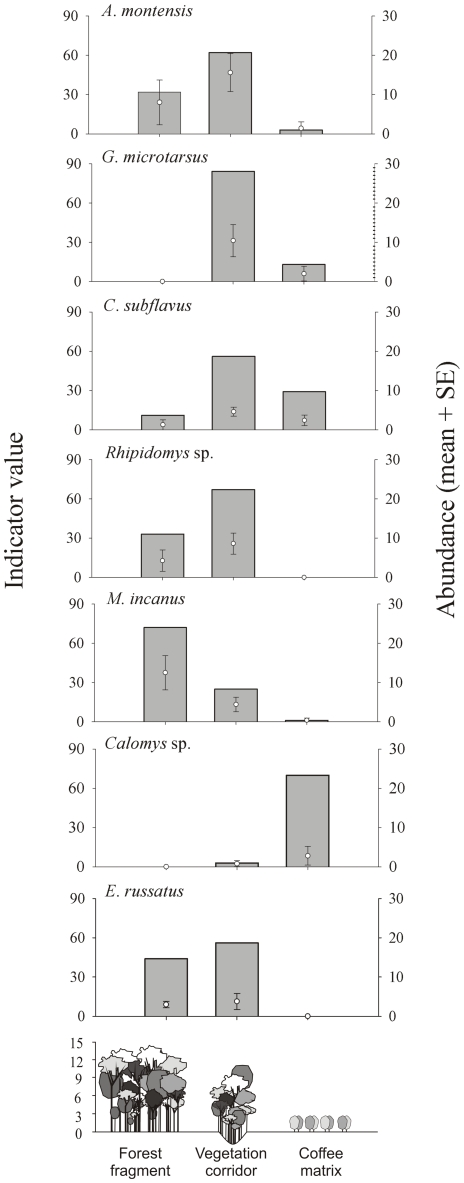
Abundance (mean and standard deviation) and indicator species values in each treatment.

**Figure 7:**
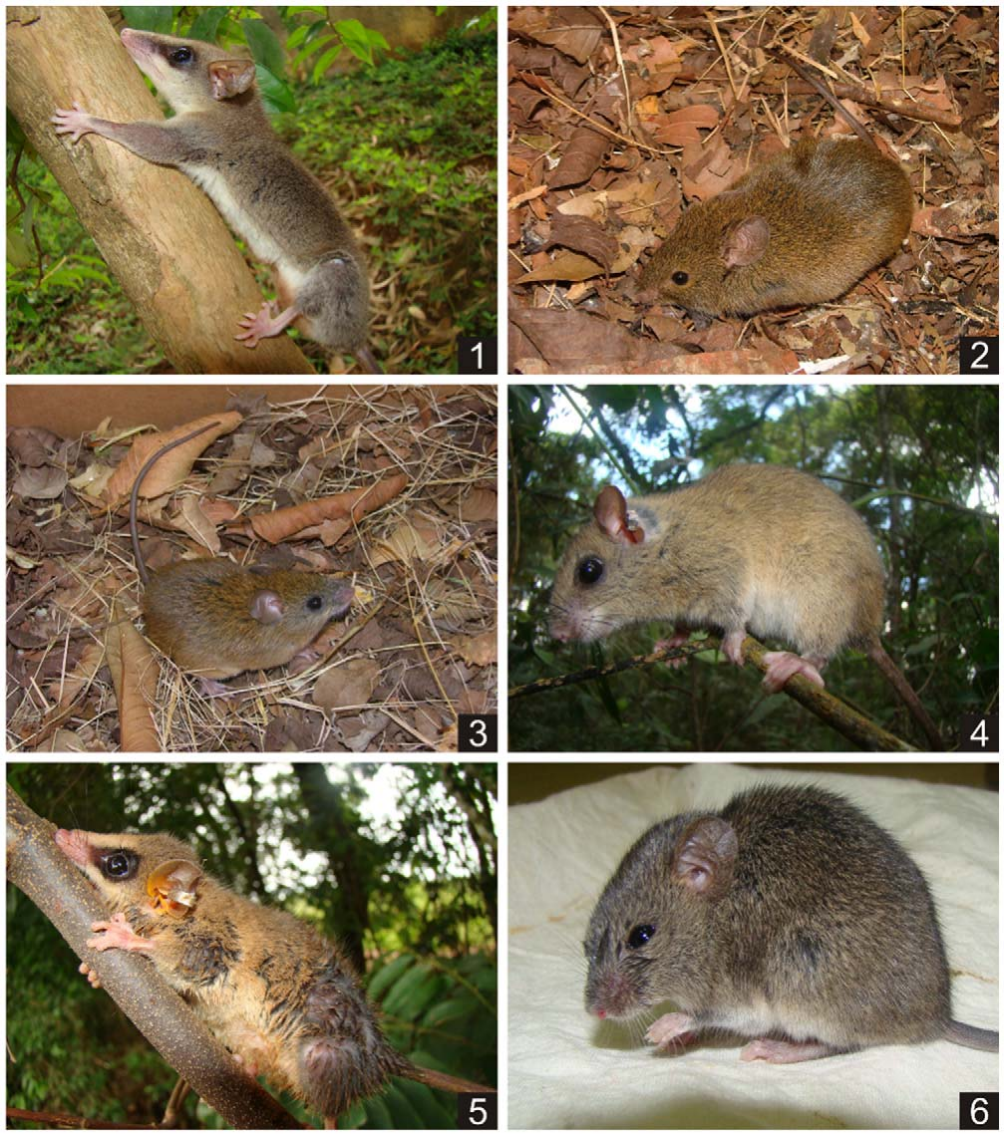
Indicator species. From left to right, (1) *Marmosops incanus*, (2) *Akodon montensis*, (3) *Cerradomys subflavus*, (4) *Rhipidomys* sp., (5) *Gracilinanus microtarsus* and (6) *Calomys* sp.

The indicator species analysis indicated that six out of the seven most abundant species had a significant preference for one of the three sampled treatments. *M. incanus* significantly preferred the fragment, *A. montensis*, *C. subflavus*, *G. microtarsus* and *Rhipidomys* sp. preferred the corridor, and *Calomys* sp. preferred the matrix. *E. russatus* indicated no significant preference for any site, presenting instead similar abundance and relative frequency values in the fragment and corridor (Figures 6 and 7).

## Discussion

The corridor sampled in this study has a narrow, linear shape and a high proportion of edge to interior, allowing its occupation by species such as *A. montensis*, *O. nigripes*, and *Calomys* sp., which were mostly associated with open sites of the nearby matrix [4], [12]. Simultaneously, the corridors provide continuity to the vegetation cover and usually are more similar to the vegetation structure in the fragment interior than that of the fragment edge [26]. This may explain the presence of species in this system shared only with the forest fragments and completely absent in the coffee matrix. The superposition of the characteristics of two distinct environments was also mentioned as a predictive factor of increasing mammal richness in the corridor in a temperate forest [32].

The lower degree of richness in the coffee matrix resulted from the low structural complexity of these sites in comparison with the corridor and fragments because small mammal species richness decreases in structurally simpler habitats [33], [34]. Most small mammals species were unable to occupy this site and are sensitive to landscape alteration. The persistence of small mammals in fragmented landscapes is strongly associated with their tolerance of open habitat surrounding fragments [4], [5], [7], [35].

Although the corridor, fragment and matrix present different compositions, the corridor was more similar to the fragments than to the matrix, indicating that this structure acts as an extension of the fragment for many small mammal species. The most abundant species in the fragment are more abundant in the corridor. The similar total abundance in the corridor and the fragments enhances the efficiency of this element in the fragmented landscape for conservation of small mammals, given that many individuals use the corridor as a habitat or for dispersion [12], [18], [19].

Even though *A. montensis*, *C. subflavus* and *G. microtarsus* displayed a significant preference for the corridor, these species occupied different treatments, corroborating available data that show that they are generally resilient to alterations of the Atlantic Forest landscape [5], [6], [11], [18], [34], [35]. *Rhipidomys* sp. seems to favor the corridor because it was unable to use the matrix. In an adjacent area exhibiting similar results, this species was common and abundant in the corridors and able to move among them [19]. *M. incanus* was significantly more abundant in the fragments than in the corridor and matrix, and as an indicator species, this marsupial reveals its sensitivity to the fragmentation of the landscape. These results reflect other studies of the Atlantic Forest indicating that this species is negatively affected by changes to the natural landscape [4], [12], [36], [37], [38].

Because *Calomys* sp. significantly prefers the matrix and shows decreasing abundance in the forest fragment, it follows that this species has more affinity for open sites, reflecting similar results for the species [19] and the genera [12], [36]. Results illustrate that habitat disturbance in fragmented forest landscapes does not affect this species. *E. russatus* displayed no significant preference for the fragment or the corridor. This species exhibits sensitivity to habitat disturbance, and results indicate that the corridor may function as an extension of its habitat, decreasing the isolation imposed by the coffee matrix, a site the species could not use.

In this region, this kind of vegetation corridor was of importance for communities of dung beetles [39], vegetation [26] and other small mammals [19]. Detour results for small mammals support these results and highlight the high conservation value of this structure in a fragmented landscape. Because these corridors occur in south and southwest Minas Gerais [26] and considering this region's intense fragmentation featuring small fragments with native vegetation immersed in agricultural lands, the results point to the necessity of specific policies that will permit the conservation of these structures on a regional scale.

The findings also indicate that the vegetation corridors function as an extension of the fragment for many species of small mammals because most fragment-inhabiting species were also present in the vegetation corridor and because the most abundant species in the fragment are more abundant in the corridor. This highlights the importance of these landscape elements in the conservation of small mammals in fragmented tropical landscapes.
